# Diabetic atherosclerosis: is there a role for the hypoxia-inducible factors?

**DOI:** 10.1042/BSR20200026

**Published:** 2020-08-20

**Authors:** Daniela Pirri, Maria Fragiadaki, Paul C. Evans

**Affiliations:** 1 Department of Infection, Immunity and Cardiovascular disease, The University of Sheffield, U.K.; 2 Singapore Bioimaging Consortium, Agency for Science, Technology and Research (A*STAR), Singapore

**Keywords:** atherosclerosis, hypoxia-inducible factors, type 2 diabetes

## Abstract

Atherosclerosis is a major cause of mortality worldwide and is driven by multiple risk factors, including diabetes. Diabetes is associated with either an insulin deficiency in its juvenile form or with insulin resistance and obesity in Type 2 diabetes mellitus, and the latter is clustered with other comorbidities to define the metabolic syndrome. Diabetes and metabolic syndrome are complex pathologies and are associated with cardiovascular risk via vascular inflammation and other mechanisms. Several transcription factors are activated upon diabetes-driven endothelial dysfunction and drive the progression of atherosclerosis. In particular, the hypoxia-inducible factor (HIF) transcription factor family is a master regulator of endothelial biology and is raising interest in the field of atherosclerosis. In this review, we will present an overview of studies contributing to the understanding of diabetes-driven atherosclerosis, integrating the role of HIF in this disease with the knowledge of its functions in metabolic syndrome and diabetic scenario.

## Introduction

Diabetes is a risk factor for cardiovascular disease (CVD), which causes around 17 millions deaths each year [1]. Diabetes is associated with myocardial infarction, heart failure and micro and macrovascular complications [2–4].

Diabetes is part of a constellation of metabolic abnormalities clinically recognised as metabolic syndrome. Metabolic syndrome is characterised by the co-existence of raised blood pressure, insulin resistance, obesity and dyslipidaemia, particularly hypertriglyceridaemia and reduced ratio of high density lipoproteins (HDL) to low density lipoproteins (LDL). Dyslipidaemia has a central role in atherosclerosis [5,6].

**Figure 1:**
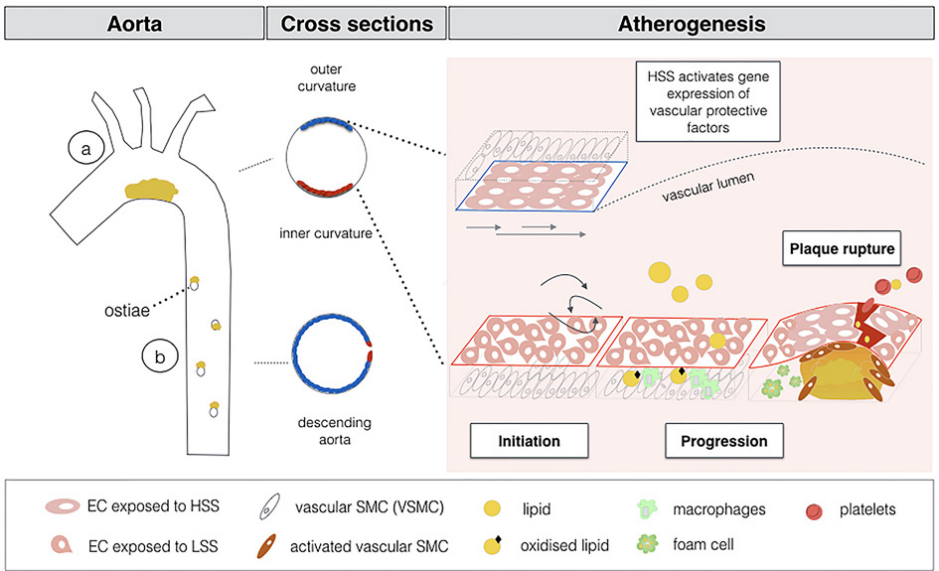
Schematic representation of site-specific atherogenesis Inner curvature of the aortic arch (**a**) is exposed to low shear stress that primes ECs for atherosclerosis (red labelled cells), while outer curvature and descending aorta (**b**) are exposed to high shear stress and ECs are protected (blue labelled cells). The three step process of atherogenesis is also represented.

Atherosclerosis is a chronic inflammatory disease characterised by the thickening of the arterial wall with the formation of a lipid-enriched plaque, as illustrated and described in Figure 1 [6]. Atherosclerosis includes initiation, progression and plaque rupture. Blood flow frictional forces (shear stress) prime endothelial cells (ECs) for atherogenesis. Atheroprone areas are found at regions exposed to low shear stress (LSS), while high shear stress (HSS) protects ECs from atherosclerosis. Atheroprone ECs allow molecules to adhere on their surface and penetrate underneath the vascular layer, initiating an inflammatory process. Lipoproteins can pass through the ECs layer and become entrapped underneath the endothelium where they are oxidised. As the inflammatory process progresses, infiltrated macrophages engulf oxidised LDL and eventually turn into large foam cells. When foam cells die the inflammatory process is exacerbated resulting in a hypoxic, necrotic and highly pro- inflammatory core. Meanwhile, vascular smooth muscle cells (VSMCs) contribute to inflammation and produce collagen to form a fibrous cap. Importantly, epidemiological studies show a direct association between hyperglycaemia and atherosclerosis [7–10]; clinical studies corroborate this link, atherosclerosis being accelerated and worsened in diabetic patients [11].

Multiple cell types contribute to the atheromatous plaque (or atheroma), and the relative abundance of these constituents differs between individuals, which in turn determines the stability of the atheroma. In humans, atheromatous plaques can break, releasing their contents into the bloodstream and activating platelet coagulation with a high risk of thrombosis. Despite the risk of a plaque partially or fully occluding the vessel lumen, thrombosis remains a major complication in plaque progression [12]. All the main cellular components of the atherogenesis, namely ECs, VSMCs and macrophages, are affected by diabetes.

Despite a large body of evidence that describe the contribution of diabetes to the development and progression of atherosclerosis, the molecular events leading to diabetes-induced atherosclerosis progression been not been revealed yet. For instance, multiple transcription factors contribute to atherosclerosis progression and some of these have key roles in controlling metabolic syndrome. This review will address the experimental evidence that underpins the role of diabetes in atherosclerosis, with a specific focus on the role of the hypoxia-inducible factor transcription factors (HIFs) in the development of diabetic atherosclerosis.

## The role of diabetes in atherosclerosis

Much of our understanding of the contribution of metabolic syndrome to the development and progression of atherosclerosis is generated by animal studies. The fundamental cross-talk between atherosclerosis and hyperglycaemia has been established in murine models employing mutation of key molecules in the regulation of the lipid metabolism, apolipoprotein E (ApoE) and low density lipoprotein receptor (LDLR), often combined with feeding of high-fat high-cholesterol diets [13–15]. To molecularly dissect the effect of hyperglycaemia and hyperlipidaemia in murine diabetic atherosclerosis, studies have used an experimental approach of pancreatic β-cell destruction in ApoE and LDLR mice thereby leading to accelerated atherosclerosis [16–19]. While it is well understood that diabetes contributes to the initial stage of endothelial dysfunction, understanding the contribution of diabetes in the progression and rupture stages of the atheroma is more complex. A major drawback is that plaque rupture is difficult to achieve in murine models [20]. Nevertheless, when the plaque is formed and diabetes is exogenously triggered, hyperglycaemia causes inter-plaque haemorrhage and destabilization at the brachiocephalic artery [21]. Therefore, suggesting that diabetes contributes to plaque destabilization. In conclusion, hyperglycaemia and hyperlipidaemia exhibit considerable cross-talk. Hyperglycaemia contributes to accelerated atherosclerosis, while hyperlipidaemia is absolutely required for developing atheroma.

## Molecular mechanisms of diabetes/atherosclerosis coupling

**Figure 2:**
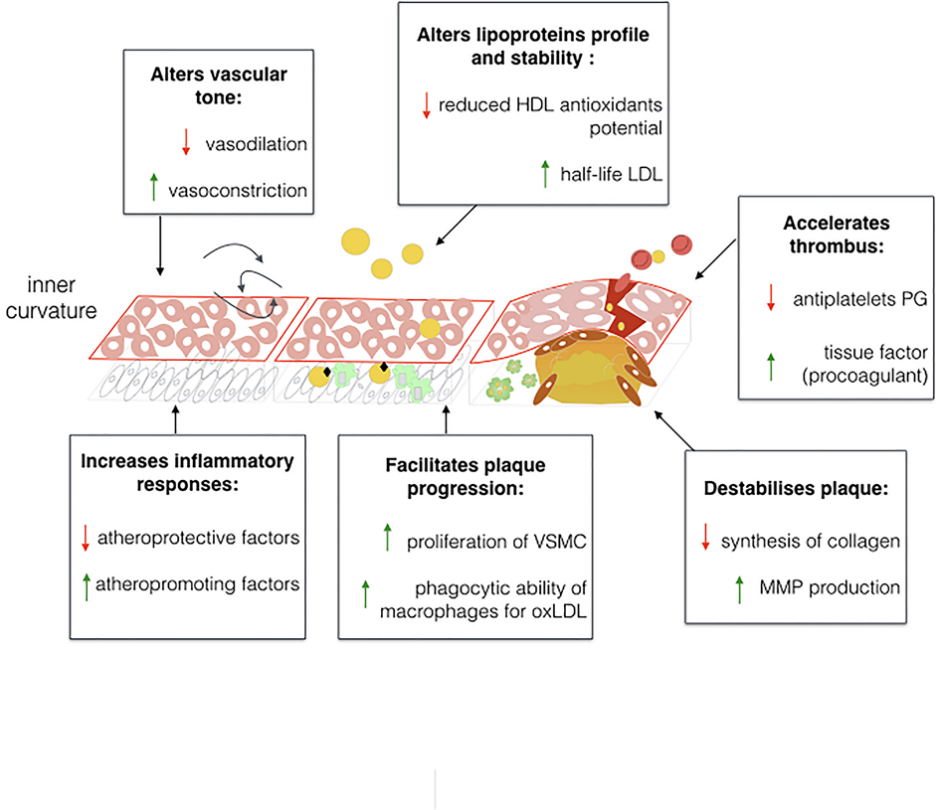
Effects of diabetes on atherogenesis Schematic summarization of some of the effects of diabetes on the vasculature. Hyperglycaemia, insulin and ROS, together with hyperlipidemia contribute to reinforce pro-inflammatory and pro-proliferative stimuli, reduce atheroprotective molecules and antioxidant defenses. High density lipoprotein, HDL; low density lipoprotein, LDL; or oxidized LDL, oxLDL; prostaglandin, PG; vascular smooth muscle cells, VSMC; matrix metalloproteinases, MMP.

Vascular homeostasis is finely tuned by both hormonal, mechanical and inflammatory factors [22]. Flowing blood with high wall shear stress confers protection to the endothelium [23]. It primes the production of vasoactive factors such as nitric oxide (NO) via, amongst other mediators, endothelial nitric oxide synthase (eNOS), which inhibits pro-inflammatory and vasoconstrictor molecules, such as the endothelin 1 (ET-1) [24–28]. On the contrary, flowing blood with low wall shear stress enhances inflammatory and proliferative responses, contributing to disease progression [29,30]. Low wall shear stress is observed at sites of ramification and bends of the aortic vessel, intriguingly these are the sites where atheromatous plaque develops preferentially [31–33]. Hyperglycaemia interacts with the endothelium exacerbating vascular disease, as shown in Figure 2. For instance, *in vitro* high glucose concentration reduces eNOS availability and reduces vascular responses to shear stress through structural rearrangement of the glycocalyx [34]. Hyperglycaemia increases pro-inflammatory activity of nuclear factor-κB (NF-κB), together with increased plasminogen activator inhibitor-1 (PAI-1) expression through diacylglycerol (DAG)-protein kinase C (PKC) mechanisms [35,36], which participates in thrombotic processes. Hyperglycaemia also contributes to endothelial dysfunction and inflammation by increasing advanced glycated end-product (AGE) formation, radical oxygen species (ROS) and increasing fatty acid oxidation and free fatty acids [37,38]. In ECs, when AGEs bind to their receptor (RAGE), they activate pro-inflammatory signalling mediated by NF-κB and increase adhesion molecules such as vascular cell adhesion protein 1 (VCAM-1).

Hyperglycaemia also contributes to atherosclerosis by altering VSMCs. The AGE/RAGE interaction promotes proliferation of VSMCs. Interestingly, soluble RAGE injection in diabetic mice suppressed atherosclerosis in a glycaemia and lipid-dependent manner [16,39]**.** Therefore, it is suggested that hyperglycaemia through activation of the AGE pathway can contribute to both initiation and progression steps of atheroma formation. At the same time, insulin has a controversial role in atherosclerosis, since it confers protection to ECs by inducing eNOS production [40]. On the other hand, insulin influences disease by inducing VSMCs proliferation [41], while macrophages lacking insulin receptors have an increased capacity for endocytosis of oxLDL, through augmented expression of the oxLDL receptor CD36 [42].

Meanwhile, diabetes associated with obesity leads to an unbalanced physiology of the leptin system. Leptin is a hormone that regulates energy balance and food intake, as well as glucose homeostasis [43,44], therefore making leptin a central regular of metabolic disease. Leptin deficient (ob/ob) or leptin receptor-deficient (db/db) murine models display obesity and transient diabetes [45]. Despite multiple studies suggesting that alteration of leptin signalling enhances atherosclerosis progression, the precise mechanism by which this occurs has not been clarified yet [39,46–51].

Furthermore, diabetes-induced activation of protein kinase-β (PKC-β) increases production of AGE from the polyol pathway and leads to accelerated atherosclerosis, reduced insulin-stimulated eNOS production and increased expression of the vasoconstrictive molecule, ET-1 [52,53]. On the other hand, inhibition of the PKC-β and PKC-θ isoforms in mice prevents atherosclerosis and improves cardiac functionality [54,55]. Therefore, insulin in vascular homeostasis is a beneficial signal in macrophages and endothelium, whereas insulin has detrimental effects on VSMCs. Thus, diabetes might also be involved in the later stages of the atherogenesis.

Taken together, glycaemia, insulin and leptin are key mechanistic components of the diabetes–atherosclerosis axis. The resultant excessive ROS activity causes abnormal ECs and VSMCs physiology. Therefore, diabetes both accelerates atheroma formation and contributes to plaque instability and thrombus formation.

## A role for HIF transcription factors in diabetic atherosclerosis

**Figure 3:**
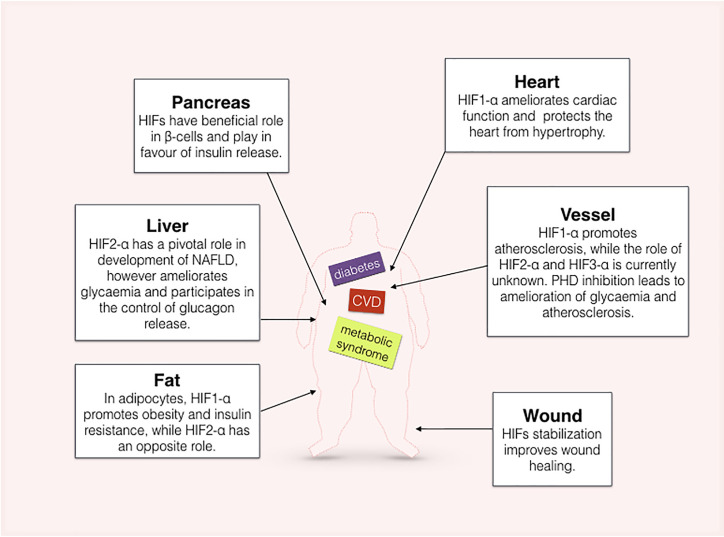
Role of HIF in diabetic organs Graphical representation of the role of HIFs at various organs.

Advanced atherosclerotic plaques presents a core with a hypoxic state, and it is associated with neovascularization and inflammatory processes that potentially contribute to plaque instability [56–58]. The molecular basis of plaque neovascularization involves the hypoxia-vascular endothelial growth factor (VEGF) axis [59,60]. Hypoxia-inducible factor (HIF) is a heterodimeric transcription factor composed of an inducible alpha subunit (HIF-α) and a constitutive beta subunit (HIF-β), also known as aryl hydrocarbon receptor nuclear translocator (ARNT) [61]. Three alpha subunits have been recognised and are differentially distributed within tissues [62]. HIFs are regulated by an oxygen-dependent prolyl hydroxylase enzymes (PHDs), thus in presence of oxygen, HIF-α subunits are rapidly hydroxylated and prepared for proteasome degradation by the interaction of ubiquitin subunits with the Von Hippel-Lindau tumor suppressor protein (pVHL) [63–65]. Whilst another oxygen-sensitive inhibitor, the factor inhibiting HIF (FIH) blocks the recruitment of 300/CBP transcriptional co-factors [66,67]. HIF is the major effector of the response to hypoxia; upon this condition, the activity of PHDs are reduced and HIF-α is stabilised and, together with HIF-β, form the transcription factor that actively binds the hypoxia responsive elements (HREs), thus activating transcriptional reprogramming of the cell [68]. There are several lines of evidence showing that diabetes and HIF pathways interact at different levels. As an example, human single-nucleotide polymorphism at the exon 12 (P582S) of HIF1-α was recurrent in Japanese patients with Type 2 diabetes mellitus [69]. A schematic summary of the action of HIFs in different organs involved in diabetes and metabolic syndrome is proposed in Figure 3.

### HIF signalling in β cells

Glycaemia is balanced by the action of two main hormones: insulin and glucagon. Within the pancreas, β cells are designated to insulin production and release with a mechanism requiring an appropriate adenosine di- and tri-phosphate (ADP/ATP) ratio. During diabetes, HIF signalling is reduced [70] along with the reduction in glycolytic enzymes; therefore, the resultant reduction in metabolic rate reduces ATP production and prevents insulin release from β cells. Thus, loss of HIF contributes to β-cells dysfunction [71]. Both *in vitro* and *in vivo* evidence suggest that HIF signalling plays a central role in β-cell function and glucose homeostasis. HIF1-α knockout in β cells impaired insulin secretion [72]. Furthermore, lack of the HIF-α isoforms counterpart, ARNT, both in mice and *in vitro* studies, showed a reduction in glycolytic enzymes [72,73]. Mice with β-cell inactivation of the *Vhlh* gene encoding for pVHL led to a marked increase in expression of genes that promote glucose uptake (e.g. glucose transporter- 1 (GLUT-1)) mediated by HIF1-α activity, and also possess impaired glucose tolerance as well as reduction in insulin levels [74]. Whilst FIH deletion in mice led to amelioration of body weight, increased insulin sensitivity, and decreased triglycerides and cholesterol levels [75]. Thus, HIF stabilisation seems to have a beneficial role in β-cell function.

### HIF signalling in diabetic adiposity and liver

Fat, metabolic disease and hypoxia signalling are intrinsically connected, as reviewed in [76]**.** Obesity and insulin resistance are observed in mice overexpressing HIF1-α [77,78]. However, adipose tissue specific HIF1-α or PHD inhibition, respectively, led to aggravation or amelioration of insulin sensitivity and obesity [79,80]. Furthermore, Lee et al. studied HIF1-α and HIF2-α in adipocytes and showed that lack of only HIF1-α or both isoforms led to reduced insulin resistance; on the contrary, solely lack of HIF2-α had the opposite effect [81]. Thus, it was thought that HIF1-α expression in adipose tissue acts in favour of obesity by leading to excess of fatty acid and triglycerides [76]. Furthermore, HIF2-α may have a more central role in regulation of obesity. It was demonstrated that HIFs transcriptionally activate the pro-opiomelanocortin (*POMC*) and that, unlike HIF1-α mRNA, HIF2-α mRNA was highly expressed in hypothalamic regions; while its protein level was enhanced after glucose or glucose metabolites infusion through the third ventricle. Loss of function of HIF2-α in hypothalamic POMC neurons favoured obesity [82].

Nevertheless, long-term hyperglycaemia and hyperlipidaemia present in diabetic patients leads to metabolic dysfunction of the liver. Diabetic patients often presented non-alcoholic fatty liver disease (NAFLD) and this pathology is associated with increased expression of HIF2-α isoform [83,84]. A model of HIF2-α stable expression by VHL and HIF1-α knockout showed that HIF2-α controls lipid metabolism in hepatocytes with reduction of fatty acid oxidation and severe hepatic fibrosis [83]. At the same time, in a model of hepatic PHD3 deletion, HIF2-α increased the expression of insulin receptor substrate 2 (IRS2), ameliorated hyperglycaemia and regulated hepatic glucose metabolism [85]. Furthermore, glucagon is the hormone responsible for counterbalancing the blood glucose levels and acts to avoid hypoglycaemia. Interestingly, transient post-prandial hypoxia in the liver activates HIF2- α and represses glucagon action by blocking the second messenger cyclic adenosine monophosphate (cAMP) [86]. Increased hepatic glucagon levels were thought to be responsible for hepatic insulin resistance in Type 2 diabetes mellitus [87]. Interestingly, patients with Chuvash polycythaemia, by VHL mutation have a modest increase in expression of HIF isoforms, and present hypoglycaemia [88]. Therefore, HIF2-α could contribute to the control glycaemia via hepatic signalling.

Despite the lack of understanding about the role of the third isoform at the moment, HIF3-α is emerging as an important regulator of body adiposity. Multiple epigenetic studies have shown a correlation between different methylation sites of the HIF3-α promoter with both adult obesity and perinatal body weight [89–91].

In conclusion, these data suggest that the three HIF-α isoforms might have different roles in obesity and metabolic syndrome. Particularly, both HIF1-α and HIF2-α might contribute to the control of glycaemic levels by their activation in pancreatic cells and hepatic cells, respectively.

### HIF signalling in diabetic hearts

HIF1-α is a crucial regulator of heart development [92–94]. In the adult heart, the following evidence revealed that HIF1-α protects myocardium. It was demonstrated that intermittent hypoxia treatment protects the myocardium from ischemia–reperfusion injury, with reduction in infarct size; mice heterozygous for Hif1-α had reduced protection when compared with their wild-type controls [95]. Cardiac overexpression of Hif1-α ameliorated and restored the level of several glycolytic and angiogenic proteins [96]. Hearts of Hif1-α heterozygous mice with exogenously-induced diabetes had decreased levels of apoptosis and altered gene expression profiles of angiogenic genes [97]. Interestingly, the level of PHD3, a key molecule of HIF protein regulation, was increased in the heart of diabetic rats and these levels were associated with increased cardiomyocyte diameter, increased apoptosis and collagen deposition, in a HIF1A independent manner [98]. Furthermore, *in vitro*, cardiomyoblasts respond to high glucose stimulation and hypoxia exposure by increasing apoptosis in a HIF1-α and Forkhead Box O3 (FOXO3)-mediated mechanism [99].

Diabetes type 2 increases fatty acid metabolism against the utilization of glucose through glycolysis; this leads to a reduction of succinate content which decreases HIF1-α levels in diabetic hearts [100]. Interestingly, during diabetes, ARNT expression is also reduced and cardiac deletion of *Arnt* in mice causes an increase in lipid accumulation and consequent cardiomyopathy [101]. Moreover, mice carrying an endothelial cell specific Hif1-α deletion driven by the Tie2 gene and subjected to transaortic constriction to induce pressure overload manifested cardiac hypertrophy and fibrosis associated with reduction of myocardial capillary density and an increase in myocardial apoptosis [102].

Overall, these data suggest that HIF1-α prevents diabetic cardiopathy; however, the roles of HIF2-α and HIF3-α in diabetic hearts are poorly studied.

### HIF signalling in diabetic vasculature and atherosclerosis

Diabetic patients have both macro and microvascular complications. The role of HIF in vascularisation in this context is evidenced by experiments where stabilisation of HIF by deferoxamine (DFO) or genetically results in improved wound-healing and angiogenesis [103,104]. Similarly, diabetic db/db mice have impaired wound healing associated with decrease in HIF signalling, and stabilisation of HIF1-α by DFO or dimethyloxalylglycine (DMOG) restores it [105]. These data suggest that HIF- driven angiogenesis may be suppressed in diabetes.

Notably, HIF proteins are major regulators of cellular glucose homeostasis, along with other essential cellular pathways (e.g. angiogenesis), HIF1-α and HIF2-α control glycolytic genes such as GLUT1 [106,107]. From a metabolic prospective, ECs have very low mitochondrial content and fatty acids or glutamine serve as alternative energetic substrates to glucose [108–111]. Therefore, ECs rely almost completely on glycolysis, either during their basal metabolism and even more during vessel sprouting [112,113]. Interestingly, the activity of 6-Phosphofructo-2-Kinase/Fructose-2,6-Biphosphatase 3 (PFKB3) promotes growth and migration of ECs and it is a master regulator of angiogenic ECs both in heath and disease [112,114].

Similarly, atheroprone areas of the vasculature are associated with a higher glycolytic rate [115,116]. Interestingly, atheroma progression is associated with endothelial-to-mesenchymal transition (EndMT) and fatty acid oxidation can protect ECs from undergoing EndMT [117–122].

Mechanosensory molecules activated by shear stress may also control metabolic enzymes. For instance, HIF1-α enhanced glycolytic enzymes in response to low wall shear stress forces, while activation of the Hippo pathway via YAP (Yes-associated protein) and TAZ (transcriptional coactivator with PDZ- binding motif) to form YAP/TAZ was demonstrated to enhance both glycolysis and glutaminolysis [115,123]. On the other hand, high wall shear stress through activation of krüppel-like factor 2 (KLF2) was suggested to inhibit glycolytic enzymes and glucose uptake [124]. Similarly, administration of the PHD inhibitor FG4497 in LDLR null mice and hypomorphism for PHD2 in a atheroprone model, conferred protection from atherosclerosis [125].

In summary, HIF1-α is a protagonist in atherosclerosis. However, its activation may be beneficial in diabetic microvascular consequences. In contrast, the role of HIF2-α and HIF3-α in atherosclerosis is much less understood. Collectively, these studies suggest that activation of the HIF family in endothelium might control metabolic rate and potentially atherosclerosis progression.

## Conclusions

In conclusion, the HIF family has diverse roles in arterial homeostasis and disease which have not been fully elucidated. HIF isoforms might have opposite or synergistic roles in vascular disease depending on context. Hence, whether HIF isoforms could be a target for therapeutic intervention of atherosclerosis is not yet clear. In this context, it will be essential to analyse the effect of single deletion of HIF isoforms on vascular homeostasis and atherosclerosis in the presence of hyperglycaemia.

## Abbreviations

ARNT: aryl hydrocarbon receptor nuclear translocator
CVD: cardiovascular disease
EC: endothelial cell
HDL: high density lipoproteins
HIF: hypoxia-inducible factor
HRE: hypoxia responsive element
HSS: high shear stress
LDL: low density lipoproteins
LSS: low shear stress
NF-κB: nuclear factor-κB
PAI-1: plasminogen activator inhibitor-1
PKC-β: protein kinase-β
ROS: radical oxygen species
VSMC: vascular smooth muscle cell
